# Supplementary material for: Mapping leadership and communities in EU-funded research through network analysis
Source: Open Res Eur. 2025 Aug 7;4:268. Originally published 2024 Dec 18. [Version 2] doi: 10.12688/openreseurope.18544.2 (PMC12421225; doi:10.12688/openreseurope.18544.2)
Supplement: Peer review history

# Reviewer report: Reviewer response for version 2

**DOI:** 10.21956/openreseurope.22476.r58795

**Published:** 2025-09-09

**Authors:** Chanel Tri Handoko

**Affiliations:** 1 Sebelas Maret University, Surakarta, Indonesia

The authors introduce a robust approach to network analysis using CORDIS data, applying AI-driven classification and community detection algorithms to reveal the evolution of project collaborations from 2015 to 2029. The North Adriatic Hydrogen Valley (NAHV) is used as a case study to highlight methodological advancements and practical insights. The work is contextualized against contemporary literature, emphasizing gaps and innovations in mapping EU research collaborations. Methodological steps, including data acquisition, network construction, centrality and coreness analysis, and consensus approaches for community detection, are clearly outlined. Results underscore the increasing scale and stability of research communities driven by EU hydrogen initiatives, with implications for policy and future research.

 1. The introduction concisely positions the study within the broader context of EU-funded innovation research, drawing from text mining and network analysis literature. The manuscript highlights prior limitations of network methods applied to CORDIS data and references several recent surveys and application papers. To improve engagement, the manuscript could discuss specific recent findings from the referenced text-mining and community detection studies in more detail, especially regarding methodological choices (e.g., how consensus community detection advances the field relative to alternative methods)

 2. The stepwise methodology is well-structured, including pseudocode and detailed algorithmic descriptions for each stage: data preparation, network construction, centrality and coreness computation, community detection, and longitudinal tracking. The choice of specific algorithms (Louvain, Leiden, Label Propagation, Edge Betweenness, Leading Eigenvector, Walktrap, Infomap) is justified, and the strengths/limitations of each are empirically explored in the results section. The technical discussion of how consensus and quality checks are implemented could be expanded, especially on algorithm selection and validation, perhaps with more concrete implementation notes or reproducibility factors.

 3. The manuscript might briefly summarize the data cleaning and validation steps performed before analysis, including potential issues with missing or inconsistent records from CORDIS.

 4. The manuscript may benefit from including summary statistics (mean, variance, etc.) for centrality measures, and quantitative measures of algorithmic performance or computational cost comparison across algorithms

If applicable, is the statistical analysis and its interpretation appropriate?

Yes

Is the study design appropriate and is the work technically sound?

Yes

Is the work clearly and accurately presented and does it engage with the current literature?

Yes

Are the conclusions drawn adequately supported by the results?

Yes

Are sufficient details of methods and analysis provided to allow replication by others?

Yes

Are all the source data and materials underlying the results available?

Yes

Reviewer Expertise:

renewable energy, public partner partnership

I confirm that I have read this submission and believe that I have an appropriate level of expertise to confirm that it is of an acceptable scientific standard, however I have significant reservations, as outlined above.

# Reviewer report: Reviewer response for version 1

**DOI:** 10.21956/openreseurope.20048.r48915

**Published:** 2025-01-07

**Authors:** Andreas V Olympios

**Affiliations:** 1 University of Cyprus, Nicosia, Cyprus

This paper develops a methodology to analyse collaborations over time among participants in Horizon 2020 and Horizon Europe, using the hydrogen energy sector as a case study. I would like to thank the authors for their interesting work, and provide my comments regarding the strengths and areas for improvement:

- The introduction begins clearly but a more comprehensive review of the literature is needed. It would be useful to include an in-depth analysis of previously used methods and their limitations before discussing the novel contributions of this specific paper.

- While hydrogen is identified as a case study, the introduction focuses almost entirely on this specific topic, which creates a disconnect. If hydrogen is just the case study, the discussion in the introduction should be broader, emphasising the general methodology rather than the specific application.
- Algorithm 1 is presented in a mixed format, combining textual descriptions and pseudocode. This could be improved by separating the process description into a table and presenting the pseudocode independently for clarity.
- The data acquisition strategy appears logical and well-thought-out.
- Captions for all figures should be detailed enough to make the figures self-explanatory. Some captions, such as for Figure 3, lack sufficient detail.

- Figure 3 requires further explanation, and the image quality appears compressed, making the text difficult to read.
- The font sizes used in figures are inconsistent. For example, compare the y-axis labels in Figures 4 and 5.
- Figure 4 timeline begins in 2015, but the caption states 2016.
- The downward trend from 2025–2029 in Figure 4 is unclear. The paper mentions a decrease in partners and collaborations but does not explain why. If the decline is due to incomplete data (e.g., new projects not yet appearing), the figure may be misleading and should be clarified to avoid confusion.

- The classification of projects into market-oriented and technology-oriented categories reveals interesting trends. However, the notable increase in market-oriented projects in 2023 is not discussed in detail, leaving a gap in interpretation.
- Figure 6 is interesting, but the small font size makes the organization names difficult to read. It would be good to increase the size or restructure the figure for clarity.
- For Figure 8, the paper mentions that one-third of the solutions are invalid according to the literature, but the supporting references or discussion are missing at this point.
- Figures 9 and 10 lack legends and are challenging to interpret. Adding legends or annotations that correspond to the organizations or data points would make them informative.

- The professionalism of the paper could be improved by ensuring consistency in figure design, font sizes, and formatting.

Overall, the paper introduces a methodology that can provide insights into collaborations in the hydrogen energy sector, but there are some areas for improvement, particularly in terms of literature review, presentation of figures, and depth of analysis.

If applicable, is the statistical analysis and its interpretation appropriate?

Yes

Is the study design appropriate and is the work technically sound?

Yes

Is the work clearly and accurately presented and does it engage with the current literature?

Partly

Are the conclusions drawn adequately supported by the results?

Yes

Are sufficient details of methods and analysis provided to allow replication by others?

Yes

Are all the source data and materials underlying the results available?

Yes

Reviewer Expertise:

Energy systems, heat pumps, buildings, green hydrogen, combined heat and power, optimisation.

I confirm that I have read this submission and believe that I have an appropriate level of expertise to confirm that it is of an acceptable scientific standard, however I have significant reservations, as outlined above.

# Response

**Published:** 2025-06-09

**Authors:** Fabio Morea

Thank you for your detailed review. Your feedback has been very helpful in identifying specific areas for improvement, and we have revised the manuscript accordingly. The updated version will be submitted shortly. Specifically, we expanded the introduction to provide a broader framing of the methodology and added a more comprehensive literature review on network analysis. Algorithm 1 has been reformatted for clarity, and several improvements were made to the figures and captions.

 Best regards,

 the Authors

# Reviewer report: Reviewer response for version 1

**DOI:** 10.21956/openreseurope.20048.r48910

**Published:** 2024-12-26

**Authors:** Laila Zemīte

**Affiliations:** 1 Riga Technical University, Riga, Latvia

The research article "Mapping leadership and communities in EU-funded research through network analysis" by Fabio Morea, Alberto Soraci, and Domenico De Stefano introduces a novel methodology for analysing collaborations and leadership roles in EU-funded research projects using network analysis. The study focuses on the hydrogen energy sector, particularly the North Adriatic Hydrogen Valley (NAHV) project, and utilizes data from the CORDIS portal, which provides comprehensive information on Horizon 2020 and Horizon Europe projects. The methodology involves downloading and enriching data from CORDIS, segmenting it by year, and transforming it into weighted networks representing collaborations between organizations.  Centrality measures are used to assess the influence of individual organizations, while community detection algorithms identify stable collaborations. Temporal analysis tracks the evolution of these roles and communities over time. The results demonstrate the emergence of key leaders and stable communities within the hydrogen energy sector, highlighting significant collaboration. The insights gained are valuable for policymakers and organizations seeking to foster innovation through sustained partnerships. The approach can be extended to other sectors, offering a framework for understanding the impact of EU research funding on collaboration and leadership dynamics. The article also discusses the concept of hydrogen valleys, which are integrated ecosystems that produce, exchange, and consume hydrogen, covering the entire hydrogen value chain. The NAHV project, involving 37 partners from Croatia, Slovenia, and the Italian region of Friuli Venezia Giulia, aims to develop a long-lasting hydrogen economy through a systemic and holistic approach. The study's findings reveal that Horizon grants in the hydrogen sector have led to the formation of stable, long-term partnerships, particularly in the domain of hydrogen valleys. The methodology presented can improve visualizations in the Horizon Dashboard and provide valuable insights for both policymakers and project coordinators. Future work will focus on monitoring the centrality measures of NAHV partners and applying the methodology to other case studies.

 The paper presents an excellent framework for analysing collaborative networks within the hydrogen sector. The paper employs advanced centrality measures and community detection algorithms to identify influential organizations and analyse the stability of collaborations over time. By focusing on the North Adriatic Hydrogen Valley (NAHV) project, the study demonstrates how EU-funded initiatives effectively foster collaboration among industry, academia, and government, while addressing critical sustainability challenges. The methodology for analysing collaborative dynamics and the entire hydrogen value chain is both innovative and meticulously detailed. It skillfully integrates technical approaches, such as network analysis, with practical applications, including policy impact evaluation. The robust data acquisition process and thorough analysis of collaboration patterns further strengthen the paper's contribution to the field.

 The article's current title does not fully reflect its focus on hydrogen projects, and its abstract lacks emphasis on the hydrogen sector. A revised title, such as **“Mapping Leadership and Communities in EU-funded Hydrogen Research Through Network Analysis”**, will better align with the paper's subject matter and objectives. Additionally, the abstract should be refined to highlight the centrality of hydrogen research in the analysis.

 Section 1.1 limits its scope by primarily discussing the NAHV project. Expanding this section to include a broader examination of existing, planned, and proposed hydrogen valleys across the EU would significantly enhance the article's value. Incorporating insights from sources like the Clean Hydrogen Partnership’s database (e.g., https://h2v.eu/hydrogen-valleys) will provide a more comprehensive overview of the hydrogen valleys landscape. This expanded approach will highlight regional variations, common challenges, and synergies, contributing to the research objectives and aligning with the EU's broader hydrogen strategy.

 The study's reliance on the CORDIS dataset, while robust, restricts its scope by excluding data from other funding sources such as local Member State calls, the Connecting Europe Facility (CEF), or the Interregional Innovation Investment (I3) instrument. To address this limitation the methodology could be enhanced by integrating data from these additional funding sources. Including insights from diverse datasets would expand the applicability of the findings, offering more actionable recommendations for policymakers and stakeholders, while capturing the full spectrum of hydrogen-related projects within and beyond EU programs. Please include details on the methodologies and techniques for integrating different datasets to encompass additional funding sources.

If applicable, is the statistical analysis and its interpretation appropriate?

Partly

Is the study design appropriate and is the work technically sound?

Yes

Is the work clearly and accurately presented and does it engage with the current literature?

Yes

Are the conclusions drawn adequately supported by the results?

Yes

Are sufficient details of methods and analysis provided to allow replication by others?

Yes

Are all the source data and materials underlying the results available?

Partly

Reviewer Expertise:

My areas of research focus on renewable energy, hydrogen technologies, energy storage, smart energy systems, electricity grid systems, risk analysis in energy, and the regulation of electricity and gas transmission. My work also includes expertise in gas infrastructure, energy security, and sustainable energy development. Additionally, I contribute to innovation in smart grids and energy market regulations.

I confirm that I have read this submission and believe that I have an appropriate level of expertise to confirm that it is of an acceptable scientific standard.

# Response

**Published:** 2025-06-09

**Authors:** Fabio Morea

Thank you for your constructive review.

 Your feedback helped us improve the structure and clarity of the article. The revised manuscript (to be submitted shortly) reflects your suggestions and incorporates recent literature. Key changes include a revised title and abstract (updated to better reflect the contents of the paper) and an expanded introduction chapter to provide a broader overview of hydrogen valleys. This has made the introduction significantly longer—hopefully not excessively so. 

 Regarding additional funding sources (e.g. CEF, I3, national/regional grants), we fully agree that this is an important direction. However, due to the differing structure, scope, and accessibility of these datasets, we see this as a separate line of work, which we plan to explore in a future study.

 Thank you again for your valuable contribution

 Best regards,

 the Authors
